# Interactive media for parental education on managing children chronic condition: a systematic review of the literature

**DOI:** 10.1186/s12887-015-0517-2

**Published:** 2015-12-03

**Authors:** Ali Annaim, Mia Lassiter, Anthony J. Viera, Maria Ferris

**Affiliations:** Department of Medicine, Division of Nephrology and Hypertension, University of North Carolina at Chapel Hill, 7021 Burnett-Womack, Chapel Hill, NC 27599 USA; Department of Family Medicine, University of North Carolina at Chapel Hill, Chapel Hill, NC USA

**Keywords:** Parent education, Interactive media, Chronic condition

## Abstract

**Background:**

Although some research has examined the use of games for the education of pediatric patients, the use of technology for parental education seems like an appropriate application as it has been a part of the popular culture for at least 30 years. The main objective of this systematic review is to examine the literature for research evaluating the use of interactive media in the education of parents of children with chronic conditions.

**Methods:**

We searched the MEDLINE, PSYCHINFO, CINAHL, Cochrane database of systematic reviews and EMBASE databases from 1986 to 2014 seeking original investigations on the use of interactive media and video games to educate parents of children with chronic conditions. Cohort studies, randomized control trials, and observational studies were included in our search of the literature.

Two investigators reviewed abstracts and full texts as necessary. The quality of the studies was assessed using the GRADE guidelines.

Overall trend in the results and the degree of certainty in the results were considered when assessing the body of literature pertaining to our focused questions.

**Results:**

Our initial search identified 4367 papers, but only 12 fulfilled the criterion established for final analysis, with the majority of the studies having flaws that reduced their quality. These papers reported mostly positive results supporting the idea that parent education is possible through interactive media.

**Conclusion:**

We found limited evidence of the effectiveness of using serious games and or interactive media to educate parents of children with chronic conditions.

**Electronic supplementary material:**

The online version of this article (doi:10.1186/s12887-015-0517-2) contains supplementary material, which is available to authorized users.

## Background

The estimated number of children with chronic health conditions in the United States is 15 to 18 million [1]. These large numbers of children rely on their caregivers for the majority of their care and health management by virtue of them being dependents. The parent/caregiver roles include learning about their child’s condition, giving medications, ensuring that the child performs procedures, and providing transportation to appointments with health care providers [2].

The number of adults, who have grown up with technology, in particular interactive media such as computer-delivered education and video games, has been rising with each successive generation. Although the use of computer-based technology and video games has been described as a means to teach children to self-manage conditions such as cancer, asthma, and diabetes, the state of the literature on the use of interactive technology and/or video games to teach parents about their children’s conditions has not been well characterized [3–9]. Such interventions have been described in the literature as “serious games” [10]. The goal of this systematic review of the literature was to identify and evaluate research that had used interactive media approaches to educate parents of pediatric patients with chronic conditions and their relative effectiveness.

## Methods

We conducted a review of publications using the following databases: MEDLINE, PsychINFO, CINAHL, and EMBASE, and we reported the findings based on the Preferred Reporting Items for Systematic Review and Meta-Analyses (PRISMA) criteria [11]. The search terms used are presented in Table 1. Additionally, the Cochrane database of systematic reviews was accessed, and an review focused on interactive health communication applications acted as a source for additional articles that the reviews examined [12]. To be included in the review, an article had to report data evaluating an educational intervention using either interactive media or interactive games for parents. Any original research design was eligible. The titles of the articles were scanned to identify pertinent studies for abstract evaluation based on the presence of an educational intervention in the title. Two authors independently examined abstracts from the most relevant articles; any disagreement between the authors was settled by a senior advisor. They also performed reference chaining using the discovered studies as a starting point. They focused on the following research question (see Table 2). The primary research question was: *Does the use of “serious games” (games intended to educate) and/or interactive media for parents improve health outcomes in children with chronic conditions (e.g. asthma, diabetes, chronic kidney disease, cystic fibrosis, and cardiac abnormalities)?*

**Table 1:** Search terms: search strategies and key words

Database	Search strategies and key words
Pubmed	“(parent education) AND (chronic condition OR diabetes OR asthma OR Chronic kidney disease OR Cystic fibrosis OR Congenital heart disease OR CANCER) AND (interactive media OR game OR interactive tool)” parent education AND (chronic condition OR diabetes OR asthma OR Chronic kidney disease OR Cystic fibrosis OR Congenital heart disease OR CANCER) AND (computer OR software)” “(parent* education OR parent learning) AND (chronic condition OR cancer OR diabetes OR CF OR congenital heart disease OR chronic kidney disease OR asthma OR chronic illness) AND (interactive media OR game* OR video games OR serious games)” parent education AND (chronic condition) (parent education) AND (chronic condition OR diabetes OR asthma OR Chronic kidney disease OR Cystic fibrosis) (serious games OR game* OR interactive media) AND (chronic illness OR diabetes OR chronic kidney disease OR cystic fibrosis OR cardi*) AND (parent*)
PsychINFO	1. Computer Games/ 2. Simulation Games 3. Games/ 4. Exp Computers/ 5. INTERNET/ 6. 4 ot 5 7. 3 and 6 8. 1 or 2 or 7 9. Video gam$.tw. 10. Computer gam$.tw. 11. Online gam$.tw. 12. Online gam$.tw. 13. Interactive gam$.tw. 14. gamer$.tw. 15. Gaming.tw. 16. Digital gam$.tw. 17. 9 or 10 or 11 or 12 or 13 or 14 or 15 or 16 18. 8 or 17 19. Double blind.tw. 20. Random$.tw. 21. Control.tw. 22. 19 or 20 or 21 23. 18 or 22
EMBASE	‘video game’ OR ‘video games’/exp OR ‘video games’ OR ‘video gamer’ OR ‘video gamers’ OR ‘video gaming’ OR videogame OR videogames OR videogamer OR videogamers OR videogaming OR‘computer game’ OR ‘computer games’ OR ‘computer gamer’ OR ‘computer gamers’ OR ‘computer gaming’ OR ‘online game’ OR ‘online games’ OR ‘online gamer’ OR ‘online gamers’ OR ‘online gaming’ OR ‘game system’ OR ‘games system’ OR ‘gamer system’ OR ‘gamers system’ OR ‘gaming system’ OR ‘game systems’ OR ‘games systems’ OR ‘gamer systems’ OR ‘gamers systems’OR ‘gaming systems’ OR ‘arcade game’ OR ‘arcade games’ OR ‘arcade gamer’ OR ‘arcade gamers’ OR ‘arcade gaming’ OR playstation OR playstations OR ‘interactive game’ OR ‘interactive games’OR ‘interactive gamer’ OR ‘interactive gamers’ OR ‘interactive gaming’ OR gamer OR gamers OR ‘game console’ OR ‘game consoles’ OR ‘gaming console’ OR ‘gaming consoles’ OR ‘digital game’OR ‘digital games’ OR ‘digital gamer’ OR ‘digital gamers’ OR ‘digital gaming’ OR ‘handheld game’ OR ‘handheld games’ OR ‘handheld gamer’ OR ‘handheld gamers’ OR ‘handheld gaming’ OR‘console game’ OR ‘console games’ OR ‘console gamer’ OR ‘console gamers’ OR ‘console gaming’ OR multiplayer OR multiplayers OR gameplay OR gameplayer OR gameplayers OR gameplayingOR ‘game boy’ OR ‘game boys’ OR ‘game cube’ OR ‘game cubes’ OR nintendo OR xbox OR mmorpg OR atari OR ‘space invader’ OR ‘space invaders’ OR ‘death race’ OR ‘pac man’ OR battlezone ORastrocade OR ‘donkey kong’ OR coleco OR tetris OR ‘super mario’ OR ‘sonic the hedgehog’ OR ‘street fighter’ OR ‘mortal kombat’ OR pokemon OR frogger OR dreamcast OR ‘grand theft auto’ ORsega:ab,ti OR pong:ab,ti AND (‘parents’/exp OR parents OR ‘parent’/exp OR parent) AND (‘education’/exp OR education OR ‘learning’/exp OR learning) “parent* AND (education/exp OR education) AND ((interactive AND media) OR interactive OR serious) AND games AND ((((chronic AND conditions) OR diabetes/exp OR diabetes OR asthma/exp OR asthma OR cancer/exp OR cancer OR congenital) AND (heart/exp OR heart) AND (disease/exp OR disease)) OR cf OR chronic) AND (kidney/exp OR kidney) AND (disease/exp OR disease)”
EBSCO	“parent* education AND (chronic conditions OR cancer OR congenital heart disease OR diabetes OR asthma)”
CINAHL	“(parent education OR parent learning) AND (games Or interactive media)” parent* AND (chronic illness OR diabetes OR ckd OR chronic kidney disease OR cardiac OR cystic fibrosis) AND (interactive media OR game* OR serious games)
Web of Knowledge	Topic = (parent* education) AND Topic = ((chronic condition OR diabetes OR asthma OR CKD OR CF)) AND Topic = (interactive or simulation) (parent* education) AND Topic = (user-computer interface) Topic = (parent education OR parent*) AND Topic = (educate OR educating) AND Topic = (parenting OR disciplining) AND Topic = (chronic condition OR diabetes OR CKD OR chronic kidney disease OR asthma)

**Table 2:** Population, intervention, comparator, outcome, time allowed for outcomes, time of search of the literature, study designs allowed (PICOTTS)

Population	Parents of children with chronic illnesses (includes asthma, diabetes, chronic kidney disease, cardiac abnormalities, and or cystic fibrosis)
Intervention	Serious/educational game; interactive media
Comparator	Parent’s knowledge about their child’s condition at baseline or in control group
Outcomes	Markers of improved management of the child’s disease; parental knowledge; disease severity; health outcomes
Time allowed for interventions effect	Up to 1 year of time allowed between intervention and post-test if applicable
Time into the past for the search	1986 was the furthest back in the literature for the search
Study designs allowed	Cohort studies; RCTs; observational studies

They determined internal validity, potential for biases, accuracy and appropriateness of the analysis and applicability used in each study. The overall quality of the study was determined through an application of the GRADE guidelines [13–19]. They also determined the literacy level of the tools used in these the studies using the Flesh-Kincaid methodology.

### Inclusion/exclusion criteria

Only observational studies, cohort studies, and randomized control trials were included in the final review. They included studies that went as far as 1986, the earliest year that this topic was introduced in the literature [20]. For cohort and randomized trials, they did not differentiate studies on the basis of time between intervention and post-testing, when applicable. The participants for the studies needed to include parents in the exposure group.

### Data extraction

The studies were examined and basic information was extracted from each study. For quantitative studies data was collected on: (1) the sample size in the study; (2) the composition of the study population; (3) the measurement tool that used in the study; and (4) outcomes. Further information on the potential for biases and analysis performed in each of the quantitative studies is presented in Additional file 1 as an evidence table.

### Critical appraisal

Two co-authors critically appraised each manuscript based on previously established criteria to assess the size of each study as well as potential biases, confounders, measurement precision, generalizability, and the meaning of the findings from the study. The studies were evaluated using the GRADE methodology, using such things as risk of bias and inconsistency, to grade the studies as very low, low, moderate, and high quality [19]. If there was a disagreement existed on the quality assessment of a study, the study was discussed with a senior advisor until a consensus was reached on the grading of the study.

### Data synthesis

Because of heterogeneity in interventions and study designs they did not attempt meta-analysis. The overall trend in the results and the degree of certainty in the results were considered when assessing the body of literature pertaining to our focused questions.

## Results

**Fig. 1:**
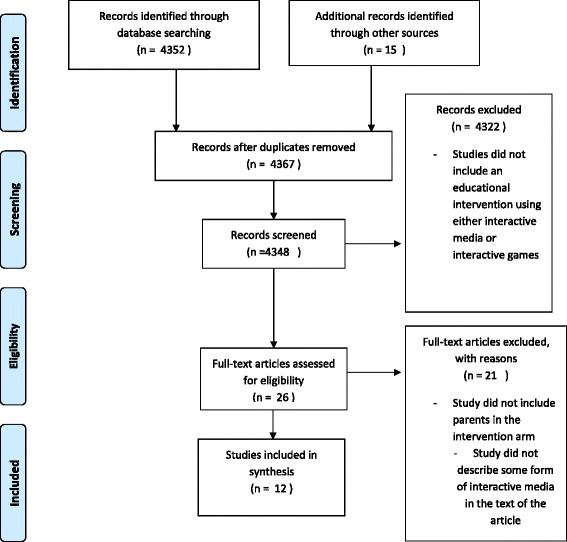
PRISMA flow diagram

Our initial search identified 4367 papers, but only 12 fulfilled the criterion established for final analysis as noted in Fig. 1. Most of the studies that explored outcomes of knowledge and/or skill showed an improvement in these outcomes after implementation of their particular intervention (Table 3). The quality of the studies found varied greatly; the majority being very poor quality studies with only one high quality study (Table 4).

**Table 3:** Synthesis table

Study authors	Population, design, intervention (tools) and Outcome	Findings	Literacy level of tools (Flesch-Kincaid Grade Level)	Assessment of Quality
Fall AJ et al. 1998 [5]	*Population*: 20 parents of children with asthma. *Design*: intervention (pre and post assessment) *Intervention*: Interactive, computer-based program; *Outcome*: Newcastle Asthma Knowledge Questionnaire	10 parents showed improved performance; 3 parents showed no change; 4 parents showed declined performance 3 dropped out Test scores improved from 21.8 at baseline to 23.5 after the intervention (95 % CI of 22.71 to 24.29) *p* = 0.06	*Intervention tool*: n/a *Outcome measure*: Newcastle Asthma Knowledge Questionnaire ranged from 2.2 to 11.9 grade level	Mixed results study; Fair quality study
Sullivan-Bolyai et al. 2012 [21]	*Populations*: Parents: (10 in pilot, 13 in focus groups and 16 in intervention) *Design*: Intervention (pre and post assessment) *Intervention*: Interactive human patient simulators *Outcome*: Change in Diabetes Awareness and Reasoning Test	For Diabetes Awareness and reasoning: 16 point increase in intervention vs 16 point increase in control, (*p* = 0.94, F = 3.15) Self-efficacy diabetes scores in intervention group increased by 8 points vs 6 point increase for control (*p* = 0.68, F = 0.17) Hypoglycemia fear-survey showed a 5 point decrease in scores for the intervention group vs 7 decrease in controls (*p* = 0.87, F = 0.03), lower scores showed less fear of hypoglycemia	n/a	The internal validity of the study was good; randomization occurred in the pilot arm to limit potential confounders but small cohort that was randomized
Thompson et al.[22]	*Population*: Inner city population at a library, a Department of Motor Vehicles office, and a fast food chain location. 20–25 % of the population in the area was under the age of 14, 49–65 % had a high school education or less for adults, and 26–32 % of the parents were single. *Intervention*: Use of an information kiosk *Outcome*: Interest in using the information provided from the sessions 1846 sessions of informational kiosk use	1447 session of the interactive kiosk (47 % at the fast food chain location; 35 % at the public library; 18 % at the DMV) 165 of the 250 respondents who completed the exit survey found the information from the kiosk useful; 113 respondents said they plan to talk with their physicians about the information from the sessions		Fair quality observational study; Skew of results from one site but large sample population. Lack of statistical analyses results for associations is concerning.
Wenninger et al. 2000 [23]	*Population*: 129 families of children with Atopic Dermatitis *Intervention*: Combination of psychological counseling in addition to clinic visits *Outcome*: Disease severity, Quality of Life of the parents, and coping skills of parents and patients	Severity of Atopic Dermatitis (SCORE-AD scores) decreased by 20.5 points in intervention vs 16.2 points in control (t = 1.27, *p* = 0.21) Confidence in medical treatment in the intervention arm (F = 7.96 of MANOVA, *P* < 0.01) Coping skills surveyed showed decreased rumination in the intervention group (t =2.44, *p* < 0.05)		Good internal validity of the study, but concerned about the lack of tables comparing the baseline characteristics of the intervention and control groups within the study
Huss et al. 2003 [24]	*Population*: children with asthma *Intervention*: Computer based asthma education program *Outcome*: Disease severity, Patient quality of life, Disease knowledge	No difference in asthma knowledge between the intervention and control group. Intervent group had a 0.4 point improvement on Air Control testing vs control groups 0.3 points improvement (95 % CI, −0.3 to 1.1) *p* >0.05) No significant changes found in pulmonary function tests. No significant change in responses to Asthma Knowledge Questionnaire	N/A	Fair internal validity for the study, but analysis did not mention controlling for asthma severity. Lack of actual statistical values for some analyses is concerning.
Guendelman et al. 2002 [25]	*Population*: 134 children with Asthma *Intervention*: An interactive health learning device *Outcome*: Limitation in physical activity, use of health services, peak flow readings	19/62 intervention and 26/60 control children had peak flow readings in yellow or red zone (OR = 0.43, (0.23,0.82) *p* = 0.01); 20/62 intervention and 28/60 control children reported limitations in physical activity (OR = 0.52 (0.29,0.94) *p* = 0.03); 4/62 intervention and 1/60 control children had a hospitalization during study period (OR = 0.99 (0.25,3.88) *p* = 0.96); 6/62 intervention and 11/60 control children had an ED visit (OR 0.59 (0.25, 1.35) *P* = 0.21)	N/A	Good internal validity with allocation concealment and randomization
Shegog et al. 2001 [26]	*Population*: 71 urban, minority children with asthma *Intervention*: Computer-based asthma education program *Outcome*: Disease knowledge, self-efficacy	Intervention group had an improvement in their knowledge scores (21.1, 95 % CI [19.38 to 22.82] *p* <0.01) Self-efficacy showed an improvement in the intervention group (mean 56.5, 95 % CI [53.38, 59.62] *p* = 0.04)	N/A	Fair study, analysis did not try to control for disease severity or child’s performance in school
Horan et al. 1990 [27]	*Population*: 20 adolescents with Diabetes *Intervention*: Computer based program to educate and monitor diabetes *Outcome*: Hemglobin A1c percentages, bloodglucose levels, disease knowledge	Disease knowledge improved in 60 % of intervention and 50 % of control children Improvement in blood glucose in the intervention group prior to lunch (F = 10.922, *p* < 0.02) and prior to dinner (F = 7.221, *p* < 0.025)	N/A	Poor internal validity, selection bias introduced through matching without controlling being performed in the analysis
Dragone et al. 2002 [28]	*Population*: 31 children with leukemia *Intervention*: CD-ROM based education program for cancer *Outcome*: Sense of control through health locus of control survey	Intervention group had an improvement in their survey results (r^2^ = 0.33, F = 6.38, *p* = 0.004)	CD-ROM program reading level 5.5.	Good internal validity study, analysis did not try to control for parental education level
Krishna et al. 2003 [29]	*Population*: 228 children with asthma *Intervention*: Internet-enabled interactive media asthma education program. *Outcome*: Disease knowledge, caregiver Quality of Life	Intervention groups showed improved disease knowledge (2.52, 95 CI [−0.38, 5.42], *p* = 0.029) Quality of Life scores showed no difference between the groups.		Fair internal validity, quality of life had a small recall interval and no attempts to control for caregiver education through analysis
Homer et al. 2002 [30]	*Population*: 106 high risk urban children with asthma *Intervention*: Multimedia software for asthma education *Outcome*: Number of ED visits and acute office visits, parent knowledge of disease, child knowledge of disease	Intervention and control groups had reduction in ED visits (2.14 to 0.86 in intervention group, 2.24 to 0.73 in control) with no statistical difference between the groups Parent knowledge (score of 80 in intervention and 78 in control) was no statistically different between the groups Child knowledge improved both groups (F =18.78, *p* < 0.001)	N/A	Good internal validity, randomization protocol was well explained and data analysis tried to adjust for possible confounders
Swallow et al. 2014 [31]	*Population*: 41 parents of children with CKD stage 3–5, 30 children with CKD stage 3–5 *Intervention*: Online parent information and support program *Outcome*: Parent management of disease, parent empowerment, father’s level of support	Parents in the intervention showed improvement in perceived competence in managing their child’s condition vs control (2.6, 95 CI (−1.6,6.7) *P* = 0.213) Intervention parents had a slight decrease in empowerment (−0.2, 95 % CI (−0.5, 0.2) *P* = 0.404) Father support level showed an decrease in score (−4.3, 95 % CI (−24.7, 16.2) *P* =0.667) among the intervention group	Online intervention Reading level 11.6	Fair internal validity, lack of adjustment for potential confounders in analysis. Possible selection bias.

**Table 4:** GRADE quality evaluation

Study	Initial quality based on study design	Factors that improve rating	Factors that worsen rating	Quality of evidence rating
Fall et al. 1998	Observational study–Low quality (2)	*Large Magnitude of effect*: Not present in this study. No reason to increase the grade (0) *Dose*–*response gradient*: Not present in this study. No reason to increase the grade (0) *All plausible confounders or other biases increase our confidence in the estimated effect*: There is a possibility that parents received education after their child had been hospitalized, which would have reduced the effect seen (+1)	*Risk of Bias*: Very serious, concern about selection process used and vague inclusion criteria (−2) *Inconsistency*: The confidence interval showed no true overlap between the pre and post-test scores. No reason to down grade since the internal consistency did not appear to be in doubt (0) *Indirectness*: Indirect measures, looking at knowledge of parents. Reasonable to downgrade (−1) *Imprecision*: Confidence Interval is not narrow and there is a small sample size. Reasonable to downgrade (−1) *Publication bias*: Small observation study, Likely to have publication bias (−1)	Very low (1)
Sullivan-Bolyai, et al. 2012	Randomized control trial: High quality (4)	*Large Magnitude of effect*: Not present (0) *Dose*–*response gradient*: Not present (0) *All plausible confounders or other biases increase our confidence in the estimated effect*: Not present (0).	*Risk of Bias*: Serious risk, lack of blinding and proper allocation concealment (−1) *Inconsistency*: Serious inconsistency, low *p*-values with small F-values, indicating possible intragroup variability (−1) *Indirectness*: Serious indirectness, study looked at self-efficacy and knowledge, no mention of patient-centered outcomes (−1) *Imprecision*: Unable to appropriately determine confidence intervals given the limited information provided in the paper. Down-grade given the small sample size and the minimal improvement in scores when experimental compared to control arm (−1) *Publication Bias*: Small pilot study, like to have publication bias since other studies with negative findings are not likely to be published. Reason to downgrade (−1)	Very low (1)
Thompson et al. 2007	Observational study–Low quality study (2)	*Large Magnitude of effect*: Not really present in this study. No reason to increase grade (0) *Dose*–*response gradient*: Not really present in this study. No reason to increase grade (0) *All plausible confounders or other biases increase our confidence in the estimated effect*: Not really present in this study. No reason to increase grade (0)	*Risk of Bias*: Serious risk of bias; concern about the fact that a majority of the data came from one location, additionally concern about the selection within the population regarding those who visit the locations where the kiosks were located (−1) *Inconsistency*: Confidence intervals that were presented were narrow, and showed an effect that was consistent. No reason to downgrade (0) *Indirectness*: Very indirect measures; looking at possibility of using the information instead of actually seeing if the information presented in the kiosks would be used (−2) *Imprecision*: Confidence intervals were narrow and consistent. Sample size is large, so it is reasonable to capture patterns. No reason to downgrade (0) *Publication Bias*: Study is rather large, and the findings are a reasonable mixture of positive and negative findings. No reason to down-grade (0)	Very Low (1)
Wenninger et al. 2000	Randomized control trial–High study quality (4)	*Large Magnitude of effect*: There was not a large magnitude of effect noted (0) *Dose*–*response gradient*: Not really present in this study. No reason to increase grade (0) *All plausible confounders or other biases increase our confidence in the estimated effect*: Not likely in this study. No reason to increase grade.(0)	*Risk of Bias*: No serious limitations, low risk of bias from some of the key areas. No reason to downgrade (0) *Inconsistency*: Results were consistent, and the statistical F values showed a reasonable effect. No reason to downgrade (0) *Indirectness*: Study employed a scale to measure disease severity, although this was not translated to direct clinical outcomes. The study also looked at quality of life and coping skills, rather indirect measures (−1) *Imprecision*: The results, although positive, showed some variability through the t-scores for the disease severity scale, which may include the change in scores seen in the control group. Consider downgrading for imprecision (−1) *Publication Bias*: No reasonable for publication bias, results showed some effect, although some of them were not statistically significant there is some clinical utility to them (0)	Low quality study (2)
Huss et al. 2003	Randomized control trial–High quality (4)	*Large Magnitude of effect*: Not present in the study. No reason to upgrade (0) *Dose*–*response gradient*: Not present in the study. No reason to upgrade (0) *All plausible confounders or other biases increase our confidence in the estimated effect*: This is possible given that since the confounding of asthma severity may cause residual biases against an effect. Reasonable to slightly rate up (+1)	*Risk of Bias*: Serious risk of bias. There was a lack of appropriate accounting of patients. There also is some selective reporting of outcomes (no real information on absolute symptom reduction). Reasonable to downgrade (−1) *Inconsistency*: The estimate of effect was consistent with findings in other studies. Additionally there is some concern about the lack of appropriate controlling for possible confounders. Reasonable to downgrade (−1) *Indirectness*: Study did try to measure disease severity through symptoms, although this is mixed in with some knowledge measures that were rather indirect. Would slightly downgrade for the indirect measures (−1) *Imprecision*: The confidence intervals are wide with some overlap between the effect seen in the intervention and the control groups. Reasonable to downgrade (−1). *Publication Bias*: The mixed nature of the results. The study was published in a reasonable journal. No reason to downgrade (0)	Very low quality study (1)
Guendelman et al. 2002	Randomized control trial- High quality (4)	*Large Magnitude of effect*: Not truly present in this study. No reason to upgrade (0) *Dose*–*response gradient*: Not truly present in this study. No reason to upgrade (0) *All plausible confounders or other biases increase our confidence in the estimated effect*: The possibile confounders of asthma severity would have worked in the direction of the effect, so there is no reason to upgrade the rating (0)	*Risk of Bias*: Low risk of bias from a few of the key criteria. There was good allocation concealment in place, although there was some loss to follow-up of some participants. No reason to downgrade (0) *Inconsistency*: No reason to downgrade. Results are consistent throughout the study, and they are similar to other studies (0) Indirectness: The study looked at disease severity, actual symptoms, ED visits, and missed days of school. These are very direct measures of the clinical effect of disease. No reason to downgrade (0) *Imprecision*: The confidence intervals for several of the odds ratios are wide. It is reasonable to downgrade for the repeatedly wide confidence intervals (−1) *Publication Bias*: No reason for possible publication bias given the thorough nature of the study (0)	Moderate quality study (3)
Shegog et al. 2001	Randomized control trial- High quality study (4)	*Large Magnitude of effect*: Not present in the study, so no reason to increase the grading (0) *Dose*–*response gradient*: No present in this study. No reason to increase the grading (0) *All plausible confounders or other biases increase our confidence in the estimated effect*: The difference between the intervention and control group, on the basis of asthma severity was not statistically significant, although this clear difference in terms of numbers would have made the intervention arm more likely to have issues with asthma, and likely more education. This confounding factor would have worked with the intervention, so there is no reason to increase the grade (0)	*Risk of Bias*: Serious risk of bias. There is concern about the use of allocation concealment in the study, as well as the randomization procedure used for the study (−1) *Inconsistency*: The results seem to be consistent throughout the study. No reason to downgrade (0) *Indirectness*: Indirect measures of knowledge were used, without any correlation to disease outcomes. Would downgrade (−1) *Imprecision*: The confidence intervals were narrow, although there is some overlap between the intervention and control groups’ intervals in the knowledge based assessments. This overlap raises some question about the imprecision (−1) *Publication Bias*: No reason to consider publication bias. The results were a mixture of positive and non-significant results (0)	Very low Quality study (1)
Horan et al. 1990	Randomized control trial with matching- High quality study	*Large Magnitude of effect*: Not present in this study (0) *Dose*–*response gradient*: Not present in this study (0) *All plausible confounders or other biases increase our confidence in the estimated effect*: The slight difference in disease knowledge at base line (more knowledge in the intervention group) would have supported the effect seen instead of working against the seen effect. No reason to increase grade (0)	*Risk of Bias*: Serious risk of bias. There is concern about the selection bias introduced through the matching process. It is reasonable to downgrade (−1) *Inconsistency*: The reasonably large F values show solid internal consistency for the study. No reason to downgrade (0) *Indirectness*: There was a direct clinically pertinent measure in this study, blood glucose levels. However, there was some indirect measures as well, knowledge and problem-solving. There is reason to downgrade (−1) *Imprecision*: The F values show some reasonable effect with slower intragroup variability. No reason to downgrade (0) *Publication Bias*: No reason to consider publication bias. The results had some reasonable support of their internal consistency through their F-values. The effect seen was small but reasonable.	Low quality study (2)
Dragone et al. 2002	Randomized control trial- High quality study (4)	*Large Magnitude of effect*: Not present in this study (0) *Dose*–*response gradient*: Not really present in this study (0) *All plausible confounders or other biases increase our confidence in the estimated effect*: The confounders that may be present would work with the effect seen in the study, so there is little likelihood that this effect would be an underestimation (0)	*Risk of Bias*: No serious risk for bias. There are slight issues with the measurement tools. No serious reason to downgrade (0) *Inconsistency*: Internal consistency seems to rather solid give the high F-values. Results are consistent with other studies, and they are supported by internal consistency. No reason to downgrade (0) *Indirectness*: Very indirect measures were used, looked at mental understanding of disease and knowledge (−2) *Imprecision*: The high F-values support the strength of the analysis that was performed. There is minimal concern for the precision of analysis (0) *Publication Bias*: The results were mixed in nature, not showing much change in the events from the interviews and modest effect for knowledge. No reason to consider publication bias (0)	Low quality study (2)
		*Large Magnitude of effect*: *Dose*–*response gradient*: *All plausible confounders or other biases increase our confidence in the estimated effect*:	*Risk of Bias*: *Inconsistency*: *Indirectness* *Imprecision*: *Publication Bias*:	
Krishna et al. 2003	Randomized control trial- High quality study (4)	*Large Magnitude of effect*: There was a small effect seen in terms of knowledge scores that improved (0) *Dose*–*response gradient*: There was no dose–response curve as all the intervention groups received the same degree of intervention. No reason to increase the rating (0) *All plausible confounders or other biases increase our confidence in the estimated effect*: One potential confounder that may be present, caregiver education, would work to minimalize the effect that is seen, so we could be slightly more confident in the effect that is seen (+1)	*Risk of Bias*: No serious concern for risk. There is some minimal concern about the measurements used, but this does not provide enough reason to downgrade (0) *Inconsistency*: Although the confidence intervals are narrow, there is overlap in several of the confidence intervals between the control and intervention groups. This raises some concern about the internal consistency of the study (−1) *Indirectness*: There was an appropriate mixture of indirect measures, knowledge scores, with clinically relevant outcomes, steroid dosage and emergency department visits. No reason to downgrade given the use of several clinically important outcomes (0) *Imprecision*: The confidence intervals that were presented appear to be sufficiently narrow. No reason to downgrade (0) *Publication Bias*: The results were mostly positive, yet the study was thorough and included clinically relevant outcomes, so the risk of publication bias seems minimal. The impact of the journal that published the study would argue that the study was rigorous evaluated. No reason to downgrade.	High quality study (4)
Homer et al. 2002	Randomized control trial- High quality study (4)	*Large Magnitude of effect*: There was not that large of an effect seen in the study. The betas that were determined did not show that strong of a relationship. No reason to increase the grading (0) *Dose*–*response gradient*: There was a slight dose–response gradient seen in some of the regression models that were performed (+1) *All plausible confounders or other biases increase our confidence in the estimated effect*: The possible confounders in the study were included in the analysis of variance. Since they were incorporated into the analysis, it seems difficult to see them working against or for the effect seen. No reason to increase the grade (0)	*Risk of Bias*: Serious risk of bias. There is some serious concern about the different sites used for recruitment, different clinical settings with possibly different patient populations served. Reasonable to downgrade (−1) *Inconsistency*: The large F-value shows some small intragroup variability to support the internal validity of the study. No reason to downgrade (0) *Indirectness*: There is a mixture of indirect measures, knowledge, and several clinical outcomes, emergency department visits and asthma severity scores. There is slightly more indirect measures, so there is some reason to downgrade slightly (−1) *Imprecision*: There is concern about the precision in the study. There is no clear way to look at regression analyses that were performed. The analysis of variance did show some reasonable intragroup study precision. Reasonable to downgrade for imprecision (−1). *Publication Bias*: There is little reason to consider publication bias. The results included some non-significant and significant findings that appeared to be appropriate. No reason to downgrade (0)	Low quality study (2)
Swallow et al. 2014	Randomized control trial- High quality study (4)	*Large Magnitude of effect*: There was not a large effect seen in this study (0) *Dose*–*response gradient*: There was not a clear dose–response gradient observed in this study (0) *All plausible confounders or other biases increase our confidence in the estimated effect*: There was the confounder of socioeconomic status, which showed the intervention group having a lower socioeconomic status. This would have worked against the effect, so our confidence that in the effect seen would be increased (+1)	*Risk of Bias*: Serious concern about selection bias introduced through the lack of blinding in the study. Also concerned about the handling of missing data in the analysis. Reasonable to downgrade (−1) *Inconsistency*: There is great variability in the intraclass coefficients presented in the study. Reasonable to downgrade for some concern about the internal consistency of the study (−1). *Indirectness*: Serious concern about the indirect measures that were used in the study, looking at the results of surveys and scales to assess parental management ability and father support (−1) *Imprecision*: The confidence intervals that are presented appear to vary widely, with overlap between the control and intervention groups. Reasonable to downgrade for precision in the study (−1) *Publication Bias*: The study was thorough, including some positive and non-significant findings. There seems to be little reason to downgrade for publication bias (0)	Very low quality study (1)

One study examined the level of knowledge that parents had regarding asthma and found an improvement in scores using the “serious game” that was presented in the study [5]. This study did not control for child’s percentage of life with the condition, so parents experience with the disease or knowledge base overtime was not evaluated (*P* = 0.06, [95 % Confidence Interval [22.71, 24.29]). The questions that were used to assess the knowledge of the participants both before and following the intervention had a standard grade level that varied greatly.

One randomized study used human simulators for improving the knowledge and management of diabetes by parents [21]. Although this was a small cohort study, improvement in self-efficacy and knowledge were promising, no statistical significance was found. This study did not include information on the child’s disease duration.

The largest study we found was an observational study that looked at the use of educational kiosks at various public locations [22]. This study’s participants found this form of interactive media to be educational; 49 % of the first time users who completed the exit survey, planned to discuss the topics presented through the kiosks with health providers. Although the study did not specify the questions used within the kiosk, there was no association between the ease of kiosk use and participant education level. Almost half of the kiosk events (47 %) came from one location (a fast food restaurant), which could have acted as a source of selection bias since participants could have different exposure level than those recruited in a library.

The next randomized study assessed the effect of intense therapy with dieticians, psychologists and providers on the self-management skills of parents whose children were diagnosed with atopic dermatitis [23]. The intervention showed improvement in medical treatment confidence by the caregivers, resulting in a greater reduction (although not statistically significant) in disease severity among the intervention participants compared to controls based on the SCORAD (a survey for scoring atopic dermatitis). This translated into a decrease of 20.5 points in the intervention arm and 16.2 points in the control arm (*p* = 0.21, t = 1.27). To measure rumination, the authors relied on the Trier scale for measuring coping with disease, but there are not actual values provided for the intervention and control arms of the study.

One randomized looked at the effects on a computer-based asthma education program on quality of life, peak flow measures, and parental knowledge [24]. There was no statistical difference between the intervention and control groups concerning asthma knowledge or asthma symptoms. There was a small improvement 0.4 in the intervention versus 0.3 in the control group in terms of their correct responses to a questionnaire related to air quality control, although this was not statistically significant (*p* > 0.05, 95 % CI [−0.3, 1.1]). This study was concerning for the lack of control for asthma severity among the intervention and control groups.

Another randomized control trial examined the effects of an interactive health learning device on children with asthma, with particular emphasis on limitations on physical activity, peak flow readings, and use of health services [25]. The study did show a difference between the groups in terms of peak flow readings (OR of 0.43, 95 % CI [0.23–0.82], *p* = 0.01) and limitation in physical activity (OR of 0.52, 95 % CI [0.29–0.94], *p* = 0.03). There was not a statistical difference between the groups in terms of hospitalizations (OR of 0.99, 95 % C [0.25, 3.88], *p* = 0.96) or emergency department (ED) visits (OR of 0.59, 95 % CI [0.26, 1.35], *p* = 0.21). The study was underpowered by the author’s own calculations, so the certainty of the effects detected is in question.

One study took a particular interest in urban, minority children with asthma in a randomization control trial of the ability computer-based education program to affect knowledge and self-efficacy [26]. The trial did demonstrate an improvement in knowledge scores of participants (knowledge scores of 21.1, 95 % CI [19.38 to 22.82], *p* < 0.01) and self-efficacy (mean 56.5, 95 % CI [53.38, 59.62], *p* = 0.04). There are concerns about the lack of controlling for disease severity or performance of children in school, which were both measured, in the data analysis.

Another diabetes focused study tested the effect of a computer based education program on blood glucose readings and disease knowledge in a match case–control trial [27]. The study did show improvement in blood glucose readings prior to lunch (F =10.922, *p* < 0.02) and prior to dinner (F = 7.221, *p* < 0.025). The lack of controlling for the factors used to match the cases and controls, as well as the small sample size, raised concerns of the internal validity of the study.

One study focused on children with leukemia assessed a CD-ROM based education program through a randomized control trial that emphasized sense of control [28]. The showed improvement in sense of control (F = 6.38, *p* = 0.004). Data analysis that did not try to control for parental education, which the authors measured, was a concern for the internal validity of the study since this factor represented a potential confounder in the development of a sense of control in the participants.

Another large study included 228 children in a randomized trial examining an Internet-based interactive media education program for asthma as it related to disease knowledge and caregiver quality of life [29]. Although there was an improvement in disease knowledge (mean change in score (2.52, 95 CI [−0.38, 5.42], *p* = 0.029) in the intervention group. The study’s internal validity was hindered by a limited recall interval for the quality of life questionnaire.

Another study of high-risk urban children with asthma assessed the ability of a multimedia education software to affect ED visits as well as parental and youth knowledge of disease [30]. The study did not observe a statistical difference in emergency department, although there was notable decrease in both groups utilization of the ED. Parental knowledge did not show a significant difference between the groups, but the knowledge of the children showed greater improvement in the intervention group (F =18.78, *p* < 0.001).

A notable study examined the effects of an online parent information support program on parental management of chronic kidney disease (CKD), parental empowerment, and the level of support from fathers concerning CKD care [31]. Parents did have an improvement in their perceived competence in disease management, although not statistically significant (2.6, 95 % CI [−1.6, 6.7], *p* = 0.213). Parental empowerment (−0.2, 95 % CI [−0.5, 0.2], *p* = 0.404) and the level of support from fathers (−4.3, 95 % CI [−24.7, 16.2], *p* = 0.667) were not significantly affected by the intervention.

The overall trend in the effects was positive in most of the studies. Only one study fit the criteria to be considered a high quality study by the GRADE guidelines [29]. Despite this lack of many high quality studies, there did appear to be a trend of positive results that showed an effect of the “serious games” on various intermediate, as well as some clinically relevant, outcomes.

## Discussion

We found limited evidence supporting the effectiveness of interactive media to educate parents of children with chronic conditions. However, a consistency of positive results supports the idea that parent education is possible through interactive media or games. The magnitude of this effect cannot be accurately determined from the studies reviewed above due to a lack of certainty in many studies, as well as varying measures that were targeted by the study designs. The effects that were shown on knowledge add to the causal linkage since parent knowledge has been associated with health outcomes in pediatric patients with chronic conditions.

It is important to understand the ability of parents to receive and process health information for the continued management of their child’s chronic condition [2]. To optimize this capacity, parental education should be tailored to their needs, culture and literacy level. It has been shown that parents go through a sense of disorientation when their child is diagnosed with a chronic condition [32]. Afterwards it may be expected that parents or caregivers will be responsible for the management of their child’s chronic condition until the patient is ready to self-manage their health.

Interactive and game platforms for parent education may be appealing because they can draw on skills that parents may have developed in their youth. These parent education platforms could also account for parental health literacy as the information could be presented with visual and auditory signals, promoting disease knowledge and management, impacting patient health outcomes. Among the studies examined in this review, only a handful demonstrated interventions that were tailored for parents with varying degrees of health literacy.

The majority of the studies showed positive and/or informative findings, which may be a reflection of publication bias in that only these findings appear to be reported. Although this is a legitimate concern, the scarcity of studies shows a gap in the literature regarding the use of interactive media for parents as learners of their child’s chronic condition. This gap represents an opportunity for improving the knowledge base of the primary care giver of children with chronic health and mental conditions. Interactive technology and/or serious games are tools that would allow parents, as well as children, to develop their self-efficacy skills in appealing formats/platforms.

From our systematic search of the literature, the studies were underpowered for the most part. This lack of large cohorts is another area that can be improved upon in order to strengthen the body of evidence. It may be argued that the lack of large cohorts makes the findings in the studies described statistically insignificant. While these small studies are not conclusive, they show trends that would be well served by being tested in larger, powered trials. Additionally, the clinical significance of findings in each of the studies, were taken into consideration. The increased knowledge that caregivers can gain from the use of interactive media has been shown to translate to decreased use of health care [33].

Another implication may be, the improved self-efficacy of the caregiver may translate to the child. One plausible sequence of events is that the child sees their caregiver gaining a handle on the information relating to their chronic illness. From there the child is inspired to learn about their illness so that they may emulate their caregiver.

## Conclusion

The literature has few studies that show improvement in intermediate measures of disease management for parents of children with chronic conditions. There were some studies that examined health outcomes, such as morbidity from disease and or health care utilization, but this area is a potential source for future research. Few large interventional studies also points to a gap in the current literature. While there does not appear to be a large amount of existing data, the evidence that is present has encouraging results.

Our findings show that interactive media could potentially serve as a tool to educate parents/caregivers about their child’s physical/mental health condition. The use of games to educate children has been explored in children with some chronic conditions [20]. It would stand to reason that parents could learn from similar games that are tailored for them. The literature at this time shows a lack of a large number of high quality studies that support this idea, and this area is one possible avenue for further research.

## Additional file

Additional file 1:
**Evidence table.** (DOCX 32 kb)

## Abbreviations

CKD: chronic kidney disease
ED: emergency department
PRISMA: Preferred Reporting Items for Systematic Review and Meta-Analyses
