# Supplementary material for: Interactive media for parental education on managing children chronic condition: a systematic review of the literature
Source: BMC Pediatr. 2015 Dec 3;15:201. doi: 10.1186/s12887-015-0517-2 (PMC4668689; doi:10.1186/s12887-015-0517-2)
Supplement: Additional file 1: — Evidence table. (DOCX 32 kb) [file 12887_2015_517_MOESM1_ESM.docx]

Additional file 1

| Author | Sample Characteristics | Sample size | Design/objectives | Main measures | Main Findings |
| --- | --- | --- | --- | --- | --- |
| Fall et al | No characteristics provided | 20 parents of children with asthma. | **Design:** intervention (pre and post assessment)  **Objective:**  Does the use of an interactive computer program improve the asthma knowledge of parents of asthmatic children? | Newcastle Asthma Knowledge Questionnaire | Mean pre-test score was 21.8, post-test score mean was 23.5 (95% CI of 22.71 to 24.29), P = 0.06; 10 of the 17 improved their knowledge after using the computer program, 3 had no change in knowledge scores, and 4 showed a decrease in their scores |
| Sullivan-Bolyai, et al 2012 | For pilot study group the mean age of parents was 42 yr, 13 female caregivers and 3 male caregivers, 15 white and 1 Latina ; mean 15.4 yrs of education | 8 in control group and 8 in intervention group | **Design:** Randomized control trial  **Objective:** explore concept of teaching parents with human patient simulator (HPS) and teach parents management education for newly diagnosed TI DM | **Diabetes Awareness and Reasoning Test-Parents**: to measure parent knowledge  **Winsock’s modified problem-solving measure**: to measure parental problem-solving abilities and knowledge synthesis  **Self-efficacy for Diabetes:** to measure parents' confidence in caring for children with diabetes  **Hypoglycemia Fear Survey**-**Parents:** to measure parental fears and avoidance behaviors associated with hypoglycemia  **State-Trait Anxiety Inventory:** to measure situational (at the tendencies and stable tendencies (general feelings) toward anxiety | Diabetes Awareness and Reasoning: experimental arm had a 16 point increase in their scores from baseline, control group had a 16 point group in their scores from baseline (p=0.94, F of 3.15)  Problem-Solving measure: experimental group had a 8 point increase in their scores, control group had a 7 point increase in their scores (F = 0.00, p = 1)  STAII - anxiety scores the experimental group had a 5 point decrease in their scores while control had a 3 point decrease in scores( lower scores meant less state anxiety, F = 0.19, p =0.67);  Self-efficacy diabetes scores increased by 8 for experimental arm and increased by 6 for control group (F = 0.17, p = 0.68,)  hypoglycemia fear-survey the experimental group had a 5 point decrease in scores while the control group had a 7 drop in scores (F= 0.03, =0.87) |
| Thompson et al 2007 | The population around this area was 20-25% under the age of 14; 49-65% had a high school education or less for adults; 26-32% parents are single. | 1846 sessions, only 1447 used for analysis | **Design:** Observational descriptive study  **Objective:**  Examine the use of touch screen health kiosks in low-income urban setting; | Poisson regressions were used to evaluate for relationship between characteristics and kiosk choices, also used from relative risk | Ease of kiosk use was associated with internet use ( prevalence ratio of 1.46 95% CI of 1.09-1.91), but not above high school education VS less ( PR of 1.04, 95% CI of 0.81-1.34)  Users with at-risk children did not vary according to grade level (p=0.72) or internet use (p=0.64)  No difference in previous users for grade level (p=0.18) or education (p=0.71) for at-risk children; previous users were more likely to view ≥2 modules (PR = 1.38, 95% CI 1.13-1.69)  Kiosk in public library had more previous users (35% of all sessions at that location) than DMV (19% and McDonalds 13%, p<0.001)  First time users at DMV were more likely to explore ≥ 2 modules (p=0.002)  Use at the 3 locations did not vary according to grade level or internet use |
| Wenninger et al 2000 | Recruited 204 families from Berlin area.  Mean age of children was 3.1 yrs (SD = 2.2), mothers ages ranged from 25-44 (mean =32.7, SD =3.9).  Mothers had a mean 11.4 years of school education (SD = 1.6 yrs), 40% had a college or university degree; 15% were single parents. | 129 cases out of 204 families recruited were completed | **Design:** Randomized control trial  **Objective:** Aimed at increasing parents' self-management skills in managing atopic dermatitis (AD) through intensive, multidisciplinary team counseling | SCORAD-score is a means of measuring AD disease  Quality of life questionnaire designed for parents of children with AD  Trier scales for measuring coping | SCORAD-scores decreased in control group by 16.2 points and 20.5 points in intervention group (t=1.27, p = 0.21).  Quality of life results differed from baseline (F = 2.2, p=0.057), marked increase in confidence in medical treatment in the treatment group found to the main reason for this change through a univariate analysis (F = 7.96, p <0.01).  Decrease in rumination through the coping styles scale was greater in intervention group (t=2.44, p<0.05) |
| Huss et al 2003 | Children with a diagnosis of asthma, age 7-12 yo, English speaking.  mean age of 9.6; 37.5 % (29/78) of intervention group and 51.1% (36/70) of control group were males; 20.8 % (16/78) intervention and and 13.3 % (9/70) in control were non-Hispanic white in intervention), 78.2% non-Hispanic black (73.2% in intervention, 84.4% in control) | 148 children recruited (78 in intervention and 70 in the control group) 101 (56 in the intervention group and 45 in the control group) had follow-up assessments; those lost to follow-up differed in age and gender; | **Design:** Randomized control trial  **Objective:** Evaluate the effects of the computer asthma education system on child's asthma symptoms, also looked at Quality of life, peak flow, knowledge, and spirometry | Air control questionnaire,  Asthma knowledge test | No difference in asthma knowledge after the two groups (initial scores were high)  0.3 point improvement in the control ( 95% CI, -0.3 to 1.1) p >0.05 and 0.4 point improvement for the intervention arm (95% CI, -0.51 to 1.11) p >0.05 on Air Control Questionnaire.  No improvement in asthma symptoms between the two groups |
| Guendelman et al 2002 | Mean age was 12 in intervention group and 12.2 in control; 60.6% (40/66) of intervention and 54.4% (37/68) of control children were male; 78.8% (52/66) of intervention and 73.5% (50/68) of controls were African-American; 92.4% (61/66) of intervention and 92.6% (63/68) of control children had Public insurance; parents were primary caregiver for asthma in 71.2% (47/66) of intervention households and 80.9% (55/68) of control households; high-school education in 39.4% (26/66) of intervention caregivers and 51.5% (35/68) of control caregivers; some college or technical school in 60.6% (40/66) of intervention caregivers and 48.5% (33/68) of control caregivers; smoking in 53% (35/66) of intervention households and 52.9% (36/68) of control households; mild asthma in 22.7% (15/66) of intervention and 29.4% (20/68) of control patients; moderate asthma 65.2% (43/66) of intervention and 58.8% (40/68) of control; severe asthma in 10.6% (7/66) of intervention and 11.85 (8/68) of control; 2.1 ED visits in past 12 mo for intervention and 2.4 vistis for control; 0.53 nights in hospital in past 12 mo for intervention and 0.66 nights for control | 136 met the inclusion criteria of the study. 96% returned for follow-up assessment (63 in the intervention arm and 65 in the control arm) | **Design:** Randomized control trial  **Objective:** Assessing if the use of an interactive learning device, Health Buddy, would decrease asthma symptoms through education of children with asthma.  Also assess if the use of the device would increase self-care behaviors, to help reduce symptoms. | Primary outcome was limitation in physical activity due to asthma  Secondary measures included days of school missed, presence of asthma symptoms, peak flow meters  Health service use measured through number of ED visits, hospital admits, or urgent calls to hospital  Self-care measured through adherence to prescribed meds and use of intervention or control (Asthma diary); | At 12 wks 19/62 intervention and 26/60 control children had yellow or red zone peak flow readings, OR of 0.43 (0.23-0.82) P = 0.01  At 12 wks, 20/62 intervention and 28/60 control children had limitations in activity, OR of 0.52 (0.29-0.94) P =0.03;  At 12 wks 39/62 intervention and 39/60 control children had cough or wheezing , OR of 0.68 (0.36-1.27), P =0.23  At 12 wks 21/62 intervention and 16/60 control children had trouble sleeping, OR of 1.07 (0.57, 2.01) P = 0.83  At 12 wks 9/62 intervention and 13/60 control children had missed school because of asthma, OR of 0.74 (0.37, 1.5) P = 0.41  At 12 wks, 6/62 intervention and 11/60 control children had ED visit in past 6 wks, OR of 0.59 (0.26,1.35) P=0.21  At 12 wks 6/62 intervention and 9/60 control children had any urgent calls to hospital, OR of 0.43 (0.18, 0.99) P =0.05  At 12 wks 4/62 intervention and 1/60 control children had been hospitalized, OR of 0.99 (0.25, 3.88) P =0.96;  Children who always used the program or asthma diary (control) were less likely to report cough or wheezing, OR of 0.70 (0.5, 0.99) and had less limitations in physical activity, OR of 0.70 (0.5, 0.9) |
| Shegog et al 2001 | Child mean age was 10.7; 65.8% ( 25/38) of intervention and 63.6% (21/33) of control males;  42.1% (16/38) of intervention and 39.4% (13/33) of control were African-American; 13.2% (5/38) of intervention and 18.2% (6/33) of control children had low asthma severity; 34.2% (13/38) of intervention and 42.4% (14/33) of control children had mild asthma; 44.7% (17/38) of intervention and 24.2% (8/33) of control children had moderate asthma; 7.89% (3/38) of intervention and 15.2% (5/33) of control children had severe asthma; 65.8% (25/38) of intervention and 81.8% (27/33) of control children made A’s and B’s; 31/6% (12/38) of intervention and 15.2% (5/33 ) of control children made B’s and C’s; 2.63% (1/38) of intervention and 3.03% (1/33) of control children made C’s and D’s; 7.89% (3/38) of intervention and 12.1% (4/33) of control children had a few times/yr experience with computers; 28.9% (11/38) of intervention and 18.2% (6/33) of control children had a few times/ mo experience with computers; 31.6% (12/38) of intervention and 36.3% (12/33) of control children had a few times/wk experience with computers; 31.6% (12/38) of intervention and 33.3% (11/33) of control children had a few times/day experience with computers | 71 children in the study with 38 in the intervention arm and 33 in the control arm | **Design:**  Randomized control trial  **Objective:**  Assess the ability of a computer based education program to teach urban, minority children with asthma  Examine knowledge and self-efficacy to asses if the computer program intrinsically motivating for children to use. | Asthma Management Questionnaire  Self-efficacy for Asthma  Attribution was measured using a survey based on the literature | Children in the intervention group had higher knowledge scores, 21.1 (19.38 to 22.82) for intervention and 17.8 ( 15.65 to 19.95), that was statistically significant (P<0.01) after controlling for pre-test scores  An outlier was found for self-efficacy score, without the outlier there was a significant difference in self-efficacy ( P=0.04 vs P=0.13 with outlier)  Total scores for asthma self-management attribution showed no statistical difference, the intervention group did have more positive attributions concerning asthma self-management (P=0.04) |
| Horan et al 1990 | 70% (7/10) of intervention group and 70% (7/10) of control group were males; 70% (7/10) of intervention group and 70% (7/10) of control group were white; average grade level was 9.9 in control and intervention group; HbA1c was 10.0% in intervention and 9.6% in control group; mean factual diabetes score knowledge score was 81.6 in intervention and 80.0 in control group; mean applied diabetes knowledge score was 81.0 in intervention and 78.7 in control group | 20 adolescents recruited from endocrinologist office and stores that sell products for diabetics | **Design:**  Matched prospect trial with randomization to control or intervention arm  **Objective:**  Asses the feasibility and general effectiveness of the Diabetes in Self-Control (DISC), a computer based program to monitor and educate DM patients | Blood glucose testing frequency  Diabetes knowledge questionnaire that was based on Test of Diabetes Knowledge: General Information and Test of Diabetes Knowledge: Problem-Solving | Intervention arm had significant increase in testing over baseline during later parts of the study ( P= 0.089, F = 3.194). Intervention group had lower values for their lunch time pre-prandial glucose measures (F = 10.922, P<0.02) than the control group. The intervention group had worse dinner pre-prandial glucoses that improved throughout the study in comparison to the control group (F= 7.221, P<0.25)  Knowledge improved in 60% of intervention and 50% of control participants  60% of intervention group reported increased activity vs 20% of control |
| Dragone et al 2002 | Mean age was 8.33 in control and 8.25 in intervention group; 80.6% (25/31) Caucasian participants, 9.68% (3/31) Latino, 3.22% (1/31)African-American, 3.22% (1/31) Asian, and 3.22% (1/31) Other race | 41 enrolled, only 31 completed the study | **Design:**  Randomized control trial  **Objective:**  Assessing if CD-ROM educational material results in greater increase in feelings of control over their health among children with leukemia, 4-6 yo and 7-11 yo, versus those using printed educational resources.  Assessing if CD-ROM education improves children’s understanding of leukemia in comparison to printed educational material.  Comparing satisfaction with CD-ROM programs versus printed education material among children with leukemia and their parents | Leukemia children’s health locus of control  Leukemia event knowledge interview | Analysis of variance was performed for pre and post-test on the health locus scale using age as a confounder ( R squared = 0.33, P=0.004, F = 6.38), for treatment group (P=0.005, F= 9.24), and for age (P=0.027, F= 5.45)  No difference between groups on pre and post-tests on the basis of number of events, number of subcategories of events from the leukemia event knowledge interviews  93.3% of intervention group found the CD easy to use vs 68.8% of the control group, who got a book (P=0.08); parents in the control group were more likely to feel that their intervention was not easy for their children to use compared to CD group (P=0.01)  73.3% of CD children used their intervention for "a long time" each time they used it compared to 12.5% of book children (P=0.0006) |
| Krishna et al 2003 | 62.8% (76/121) of control and 67.3% ( 72/107) of intervention children were male; 84.3% (102/121) of control and 86.9% (93/107) of intervention children were white; 7.43% (9/121) of control and1.87% ( 2/107) of intervention children were African-American | Recruited 246 children and their caregivers (initially 127 in control and 119 in intervention); 3 families declined (too busy, not interested, or too much hassle); 6 control and 11 intervention subjects were excluded at request of participant or lack of data; 1 intervention child was excluded because of suspected Munchausen by proxy | **Design:**  Randomized control trial  **Objective:**  Determine whether the addition of an interactive multimedia asthma education program (on top of traditional clinic-based patient education) would improve child and caregiver knowledge and health status and decrease health-care utilization; | Pediatric asthma care knowledge survey  Pediatric asthma caregiver Quality of Life survey  Pediatric asthma Quality of Life survey for children > 7 yo  Medical resource utilization through ED visits and steroid dose used | Intervention group showed an increase in knowledge; control, 2.52 (-0.38,5.42) P = 0.0293 vs 7.97 (5.00,11.00) P<0.001 for the intervention caregivers of 0-6 yo; 2.38 (0.0,4.00) P=0.079 in control vs ; 4.62 (2,7) P<0.001 in the intervention for caregivers of 7-17 yo; 4.44 (2.00,7.00) P=0.001 in control vs; 10 (7,11) P<0.001 for 7-17 yo children)  Asthma knowledge score increase in 7-17 yo children had correlated with fewer urgent physician visits (r=0.37, P=01), and decreased use of rescue medicine (r=0.3, P<0.05); intervention group had greater decrease in days with asthma symptoms (81 vs 51 decrease, P<0.01); intervention group had greater decrease in ED visits (1.93 vs 0.62 point decrease, chi-squared of 5.07, P<0.01); lower inhaled corticosteroid dose in intervention group (434 microgram vs 754micrograms, chi-squared of 7.31, P<0.01)  Children 7-17, 81% found program easy to use, 31% found it interesting, 23% found it enjoyable, 62% said they would use it again; for caregivers of 7-17 yo children 82% found it easy to use, 68% found it interesting, 41% found it enjoyable, 86% said they would use it again; for caregivers of children 0-6 yo 89% found it easy to use; 44% found it interesting, 33% found it enjoyable, and 67% said they would use it again |
| Homer et al 2002 | 29.5% of control ( 18/61) and 31.6 % of treatment (24/76) were female; 55.7% (34/61) of control and 64.5% (49/76) of treatment were black; 14.8% (9/61) of control and 13.1% (10/76) of intervention had private insurance; age at first asthma diagnosis was 2.9 in control and 3.3 in treatment arm; smokers were present 39.3% ( 24/61) of control and 40.7% (31/76) of treatment homes; 36.2% (22/61) of control and 27.6% (21/76) of treatment group missed 3 or more days of school because of asthma; ED visits in past year was 0.75 for control and 0.86 for treatment group; asthma severity was 1.05 in control and 1.11 in treatment (based on NIH criteria) | 137 parents consented to participate, 106 completed exit questionnaire; protocol required 3 visits, 61% of enrolled families returned for >1 visit, 21.3% (13/61) of control families and 31.6% (24/76) of treatment families returned for second visit | **Design:** Randomized control trial  **Objective:** To assess if a computer based asthma education program would improve asthma-related knowledge, skills and asthma-related morbidity in a high-risk urban population. | Primary outcome was number of ED visits and acute office visits  Asthma severity measured with 3 point scale (mild, moderate and severe) based on National Asthma Education and Prevention Program guidelines  Child Health Questionnaire used to measure functional status  Used a 5-point scale to measure satisfaction  Parent knowledge tested with true false questions from the National Heart, Lung, and Blood Institute Asthma IQ test  Game efficacy measured with a 13-item asthma knowledge | 106 completed the exit questionnaire (49/61 control and 57/76 intervention)  ED visits during study 2.24 to 0.73 (control) and 2.14 to 0.86 (treatment), decline in both groups with no statistical difference between the groups; acute office visits during study 0.96 to 0.77(control) and 0.91 to 0.93(treatment), no statistical difference between the groups;  Asthma severity 1.05 to 0.78 (control) and 1.11 to 0.94 (treatment), no statistical difference between the two groups, F = 5.74, P=0.018 (before and after comparison).  Parent knowledge of asthma 78 (control) and 81 (treatment) no statistical difference between the group, no baseline data on this measure; child asthma knowledge 57 to 63 (control) and 60 to 77 (treatment), F of 18.78, P<0.001;  All children in the treatment group enjoyed the game, children 8 and older were able to master skills require to operate the game after some coaching; majority of parents felt uncomfortable playing the game; parents enjoyed the educational videos |
| Swallow et al 2014 | 50 % (11/22) of parents in control and 57.9% ( 11/19) of parents in intervention group were female; 72.7% (16/22) of parents in control and 84.2% (16/19) of parents in intervention were White- European, 27.3% (6/22) of control and 10.5% (2/19) of intervention parents were South Asian; mean child age was 10.2 in control and 9.1 in intervention; 22.7% (5/22) of control and 21% (4/19) of intervention children were female; CKD stage 3 in 18.8% ( 3/16) of control and 42.9% (6/14) of intervention; CKD stage 4 in 31.2% (5/16) of control and 0/14 of intervention children; CKD 5 stage in 50% ( 8/16) of control and 57.1% of (8/14) intervention children; control group had higher level of deprivation (based on postal code neighborhood ranking) compared to intervention (13041.5 in control zipcode vs 21547 treatment , higher number = lower number of deprivation) | 25.5% (14/55) of participants dropped-out, 7 from control and 7 from intervention; control parent drop-outs were younger (37 vs 44.1 yo), intervention parent drop outs (41.4 yo vs 42.7 yo in those who remained); lower socioeconomic status in both group drop-outs; CKD stage 3 retained in control 50 % (5/10) vs 80% (8/10) in intervention; CKD stage 4 or 5 retained in control 89.5% (17/19) vs 68.8% (11/16) in intervention | **Design:** Randomized control trial  **Objective:** To assess the feasibility of a full scale RCT of their OPIS ( online patient information and support) in terms of recruitment, retention of parents, data collection procedures and psychometric performance of measures; investigate changes in outcome measures | Parent health literacy measured through the REALM  Parent management ability measured through family management measure (FaMM),;  Parent empowerment measured with the service system subscale of the family empowerment scale (FES),;  Father support was measured with the Dads active disease support scale (DADS), | For family management measure, intervention had a -0.9 [(-4.3, 2.5) P = 0.576 with r2 of 0.856] change in child's daily life scale, a 2.6 [(-1.6,6.7) P = 0.213 with r2 of 0.823] change in condition management ability scale, a 3.7 [(-4.9, 12.2) P =0.176 with r2 of 0.613] change in family life difficulty scale, a 3.8 [(-0.3, 7.9) P = 0.066 with r2 of 0.421] change in parent mutuality scale, a 0.7 [(-3.8,5.1) P = 0.763 with r2 of 0.953] change in view of condition impact; for Family empowerment scale, intervention had a -0.2 [(-0.5, 0.2) P =0.404 with r2 of 0.803] change in service system subscale; for dads' active disease support scale the intervention group had a -4.3 [(-24.7, 16.2) P = 0.667 with r2 of 0.794] change in amount score, a 12.3 [(0.9, 23.70 P =0.036 with r2 of 0.211] change in helpfulness scale; article mentions that baseline, members of the control group had the four lowest REALM scores, they had data on 36 parents at baseline and 20 weeks and 31/36 parents scored above 60 at both time points (indicates adequate level of health literacy) |
